# Evaluation of digital vaccine card in nursing practice in vaccination room*

**DOI:** 10.1590/1518-8345.3058.3225

**Published:** 2019-12-05

**Authors:** Jéssica Pereira Lopes, Thiago Magela Rodrigues Dias, Dárlinton Barbosa Feres Carvalho, Jhonatan Fernando De Oliveira, Ricardo Bezerra Cavalcante, Valéria Conceição De Oliveira

**Affiliations:** 1 Universidade Federal de São João del-Rei, Campus Centro-Oeste Dona Lindu, Divinópolis, MG, Brazil.; 2 Fundação de Amparo à Pesquisa de Minas Gerais (FAPEMIG), Brazil.; 3 Centro Federal de Educação Tecnológica de Minas Gerais, Divinópolis, MG, Brazil.; 4 Universidade Federal de São João del-Rei, Departamento de Ciência da Computação, São João del-Rei, MG, Brazil.; 5 Universidade Federal de Juiz de Fora, Departamento de Odontologia, Governador Valadares, MG, Brazil.; 6 Scholarship holder at the Coordenação de Aperfeiçoamento de Pessoal de Nível Superior (CAPES), Brazil.

**Keywords:** Nursing Informatics, Vaccination, Information Systems, Mobile Applications, Information Technology, Software, Informática em Enfermagem, Vacinação, Sistemas de Informação, Aplicativos Móveis, Tecnologias da Informação, Software, Informática Aplicada a la Enfermería, Vacunación, Sistemas de Información, Aplicaciones Móviles, Tecnología de la Información, Programas Informáticos

## Abstract

**Objective:**

develop and evaluate a vaccine application for mobile devices, with update integrated with the National Immunization Program Information System, for care in vaccination rooms.

**Method:**

methodological research based on the Pressman System Development Life Cycle theory developed in three stages: integrative literature review, computational development, and application evaluation. The product was evaluated as to satisfaction, using a validated questionnaire, and as to usability by the System Usability Scale.

**Results:**

the application functionalities were based on the survey of technological Innovations on immunization, published in the scientific literature. It displays user vaccines directly from the National Immunization Program Information System, notifies about upcoming vaccines, and enables the inclusion of vaccine cards of dependents. The evaluation resulted in users’ mean score of 90.5 ± 11.1 and health professionals’ mean score of 84.2 ± 19.4.

**Conclusion:**

the application is a technological tool with potential to improve the work process in vaccination rooms and to reach the goals of vaccine coverage. It synchronizes data with the National Immunization Program Information System, thus enabling the maintenance of people’s vaccination history.

## Abstract (pt)

**Objetivo:**

desenvolver e avaliar um aplicativo de vacina para dispositivos móveis, com atualização integrada ao Sistema de Informação do Programa Nacional de Imunização, para o cuidado em sala de vacinação.

**Método:**

pesquisa metodológica fundamentada na teoria ciclo de vida de desenvolvimento de sistemas de Pressman que foi desenvolvida em três etapas: revisão integrativa da literatura, desenvolvimento computacional e avaliação do aplicativo. O produto foi avaliado quanto à satisfação, utilizando um questionário validado, e quanto à usabilidade pelo System Usability Scale.

**Resultados:**

as funcionalidades do aplicativo basearam-se no levantamento de inovações tecnológicas sobre imunização, publicadas na literatura científica. Exibe as vacinas do usuário diretamente do Sistema de Informação do Programa Nacional de Imunização, notifica próximas vacinas e possibilita a inclusão de cartão de vacina de dependentes. Avaliação com pontuação média dos usuários foi de 90,5 ± 11,1 e dos profissionais de saúde de 84,2 ± 19,4.

**Conclusão:**

o uso do aplicativo é uma ferramenta tecnológica com potencial para melhorar o processo de trabalho em sala de vacinação e para o alcance das metas de cobertura vacinal. Sincroniza dados do Sistema de Informação do Programa Nacional de Imunização possibilitando a manutenção do histórico vacinal das pessoas.

## Abstract (es)

**Objetivo:**

desarrollar y evaluar una aplicación de vacuna para dispositivos móviles, con actualización integrada al Sistema de Información del Programa Nacional de Inmunización, para el cuidado en sala de vacunación.

**Método:**

investigación metodológica fundamentada en la teoría de ciclo de vida de desarrollo de sistemas de Pressman que fue desarrollada en tres etapas: revisión integrativa de la literatura, desarrollo computacional y evaluación de la aplicación. El producto fue evaluado cuanto a la satisfacción, utilizando un cuestionario validado, y cuanto a la usabilidad por el System Usability Scale.

**Resultados:**

las funcionalidades de la aplicación se basaron en la recopilación de innovaciones tecnológicas sobre inmunización, que fueron publicadas en la literatura científica. Exhibe las vacunas del usuario directamente del Sistema de Información del Programa Nacional de Inmunización, notifica próximas vacunas y posibilita la inclusión de tarjeta de vacuna de dependientes. Evaluación con puntuación media de los usuarios fue de 90,5 ± 11,1 y de los profesionales de salud de 84,2 ± 19,4.

**Conclusión:**

el uso de la aplicación es una herramienta tecnológica con potencial para mejorar el proceso de trabajo en sala de vacunación y para el alcance de las metas de cobertura de vacunación. Sincroniza datos del Sistema de Información del Programa Nacional de Inmunización posibilitando el mantenimiento del historial de vacunación de las personas.

## Introduction

Vaccination is an important public health instrument in the prevention and control of immunopreventable diseases and, therefore, keeping the vaccine card updated and accessible is essential to enable its benefits. However, keeping this card updated is hindered by many barriers, such as lacking knowledge about the importance of immunobiological administration, forgetting scheduled doses, and fearing possible complications related to the vaccine^(^ 1 ^-^ 2 ^)^. 

In the routine of health services, the vaccine card is presented as a paper document maintained in various types of formats and contents. This may result in problems such as loss of card and difficulty in providing the health professional with access to consistent and reliable information, as these cards are vulnerable to damage, which compromises their validity^(^ 3 ^)^. One of the ways to solve the problems concerning the maintenance of vaccination records would be the use of mobile devices^(^ 4 ^)^.

International studies highlight the use of mobile devices comprising electronic and reliable records of people’s vaccination history, providing updated vaccination schedule and reminders of future vaccines, thus contributing to improve vaccination coverage rates and organization of the immunization schedule^(^ 4 ^-^ 8 ^)^. In Brazil, a mobile device developed with the objective of permanent education of professionals and health education in vaccination was traced in the scientific literature^(^ 9 ^)^. 

Vaccination applications function as a digital vaccination card, registering vaccines and providing information to people^(^ 10 ^)^. However, a limitation in the use of applications is the reliability of the information provided. Most applications do not automatically update vaccination card records directly from the Immunization Information System (IIS), which makes it difficult to maintain the card because the person needs to manually register their vaccine records, and may compromise the validity of the information. Accordingly, as a criterion for validating this information, applications should be “linked” to the IIS^(^ 8 ^,^ 10 ^-^ 11 ^)^. Integration with the IIS enables the reliability of vaccine information for the health team and guarantees the digital vaccine card as a document to prove the person’s vaccination history^(^ 8 ^)^. 

In Brazil, traditionally, nursing assumes the entire work process in the vaccination room. Thus, a digital vaccine card, synchronized with the National Immunization Program IIS, will enable the management of care in vaccination rooms, with decision making about the vaccination situation of the person resulting in safe care, both for the nursing staff and for the person to be vaccinated. Moreover, it may enable greater involvement of the population with issues related to vaccination. 

In this perspective, this study aimed to develop and evaluate a vaccine application for mobile devices, with update integrated with the National Immunization Program Information System, for care in vaccination rooms.

## Method

This is a study of methodological development of a digital vaccine card in mobile technology, with update integrated with the SIPNI, in the form of an application called Vaccination in the Palm of the Hand (Vacinação na Palma da Mão), based on the Pressman System Development Life Cycle theory^(^ 12 ^)^. The application was developed in partnership with professors of the programs in Nursing and Computer Science of the Federal University of São João del Rei - UFSJ and with professors and students of the Federal Center of Technological Education of Minas Gerais - CEFET-MG. 

The research was carried out from March 2017 to August 2018, in a municipality in the state of Minas Gerais. The municipality’s health care network is composed of twelve health establishments, of which 7 Primary Health Care Units (PHCU), totaling a 100% coverage of the Family Health Strategy. The population estimated for 2018 was 27,755 thousand inhabitants^(^ 13 ^)^. Implementation of the SIPNI in PHCU vaccination rooms began in 2013 and, at the time of this research, the municipality had dense vaccine database registered in the system, that being the reason for its choice. All 7 PHCUs participated in the study. 

The methodological course of the present study was developed in three phases: 1st phase - integrative literature review to trace applications for mobile devices and their use in vaccination care, in order to support the construction of the application functionalities; 2nd phase - development of the computational system carried out in cycles, considering the stages of communication, planning, modeling, construction and delivery belonging to the software engineering evolutionary model, whose main characteristic is the delivery of increasingly complete versions at each iteration^(^ 12 ^)^; and 3rd phase - evaluation of the application. 

The integrative literature review traced technological innovations, their functionalities, benefits and limitations. In the analysis, the gold standard for a vaccination application was defined as: synchronizing with the IIS database, sending reminders of alert on scheduled vaccines and/or delayed vaccines, and disseminating information about immunization. The review and synthesis of knowledge were performed in the databases PUBMED, MEDLINE, LILACS, SCIELO, WHOLIS, ACM (Association for Computing Machinery) and of the Institute of Electrical and Electronic Engineers (IEEE), based on the Descriptors in Health Sciences (DeCS): Vaccination; Medical Informatics; Immunization Programs; Immunization; Mobile Applications; Cell Phones; Electronic Health Records; Technology; Vaccine e Clinical Decision Support. The inclusion criteria used for sample selection were: publications available online in English, Portuguese and Spanish, regardless of the year of publication, which addressed the IT for mobile devices in vaccination care. Theses, monographs, abstracts and review articles were excluded. 

The digital vaccine card computer system for mobile devices consisted of two parts: a) the SIPNI data synchronization system, called the Vaccination in the Palm of the Hand Synchronization System (Sistema de Sincronismo Vacinação na Palma da Mão - VPM-Sinc); b) the mobile application called Vaccination in the Palm of the Hand (Vacinação na Palma da Mão).

The development was carried out considering the software engineering evolutionary process model. The evolutionary process flow occurs in a cyclical way and presents a characteristic that enables the delivery of increasingly complete versions of the software^(^ 12 ^)^. Thus, the digital vaccine card was built in three development cycles. The first cycle aimed at developing the VPM-Sinc Synchronization System; the second cycle, at analyzing and designing interfaces of the Vaccination in Palm of the Hand application; the third cycle, at the final construction of the application in Android and iOS mobile environment and its integration with the VPM-Sinc.

The application construction considered the initial interaction between those involved in the study, including health professionals working in vaccination rooms, potential users, and researchers, aiming to meet the criteria of data security, ease of use and benefits for people and health professionals. In the planning stage, we designed the functionalities and chose tools to be used in the development cycle. The modeling stage consisted in the preparation of diagrams, which were used in the next step, in the construction of the proposed mobile application. After the construction of the functionalities, three researchers of the vaccine area checked and monitored them in order to validate their compliance with the requirements. This cycle continued until the end of development, when the application was analyzed and evaluated.

**Figure 1:**
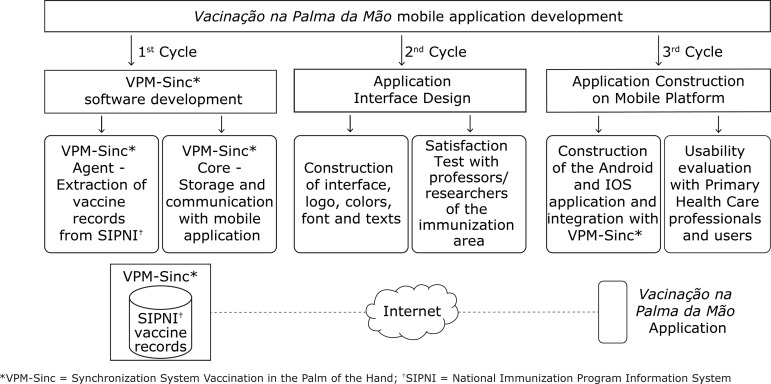
Structure of construction of the “Vacinação na Palma da Mão” mobile application *VPM-Sinc = Synchronization System Vaccination in the Palm of the Hand; †SIPNI = National Immunization Program Information System

Initially, to enable the application to update with SIPNI vaccine records, we developed the synchronization system called VPM-Sinc, which is composed of two modules (Figure 1). The first module was developed in desktop environment - for selection of SIPNI vaccine records and storage in an online database -, in Java, due to its portability, which enables installing the software in vaccination rooms with any computer operating system. The second module, developed in web environment, enables the mobile application to update with SIPNI vaccine records stored in the online database.

The application was evaluated in two stages and with three different groups: professors/researchers, nursing professionals working in the vaccination room, and primary health care users. 

For evaluation of the first version of the Vacinação na Palma da Mão application, consisting of the main interfaces of the application and proposal of integration to SIPNI, we invited seven professors/researchers that teach immunization content in the undergraduate programs in nursing and medicine of the UFSJ. All professors agreed to participate in the study. 

In April 2018, the final version of the application was evaluated, both by professionals working in the vaccination room and by users of Primary Health Care Units - PHCU of the municipality. The sample of the population was intentional and non-probabilistic. During a one-week period, we selected users served in PHCUs in the study municipality. For selection, we considered as inclusion criterion any adolescent, adult or elderly person who had a mobile device of the smartphone type and voluntarily accepted to participate in the study. The age group considered for adolescent users was 10-19 years; for adults, 20-59 years; for seniors, 60 years or older - according to Brazil’s 2018 National Vaccination Calendar, made available by the Brazilian the Ministry of Health. Nine users invited, who met the inclusion criteria, did not want to participate in the study. 

For selection of professionals, we included all those of the nursing team who worked in the vaccination room and were present in the unit at the time of data collection. Of the total 16 professionals of the municipality, 13 were present in the health unit and only one nurse did not agree to participate in the study.

In the evaluation with the professors, we performed a satisfaction test, which consisted of applying a satisfaction questionnaire^(^ 14 ^)^ that presents questions about the usability of the application, such as: ease of use; organization of information; layout of the screens; nomenclature used in the screens; system messages, assimilation of information; general concept in relation to the applied test. The questions present a numerical scale from 0 to 5, and 5 indicates the highest level of satisfaction, while 0 presents the lowest level of satisfaction. For a better understanding, words are placed in the ends (such as difficult-easy; bad-good; confused-clear; interesting-monotonous). The main objective was to collect information to deepen the understanding of the application, trace functionalities in conformities and points to be improved.

When evaluating the final version of the application, participants used the application on a mobile device, exploring its functionality more than once. After becoming familiar with the presented content and its structure, they answered the System Usability Scale - SUS^(^ 15 ^)^ questionnaire, validated in Portuguese in 2010^(^ 16 ^)^. The SUS questionnaire contains ten questions, totaling 100 points, which allow obtaining people’s overview about the system. The SUS measurement scale is of the Likert type, whose score ranges from 1 to 5 points. Participants are asked to answer whether or not they agree with the statements using the options: totally disagree (1 point), disagree (2 points), neither agree nor disagree (3 points), agree (4 points), and totally agree (5 points). It is possible to recognize, in the questionnaire, quality components such as ease of learning (questions 3, 4, 7 and 10), efficiency (questions 5, 6 and 8), ease of memorization (question 2), minimization of errors (question 6) and satisfaction (questions 1, 4 and 9).

After collecting data of the satisfaction questionnaire^(^ 14 ^)^, applied to professors/researchers, we calculated the mean score for each question answered.

The calculation of the *SUS* score is the final score obtained, calculated by means of the individual sum of the answers. For items 1, 3, 5, 7 and 9, the individual score is the received score minus 1. For items 2, 4, 6, 8 and 10, the contribution is 5 minus the received score. The sum of all scores is multiplied by 2.5, thus obtaining the total SUS value that classifies the system’s usability^(^ 15 ^)^. 

After assigning and calculating the score, it is possible to classify the evaluated system: 0 to 50 (not acceptable); 50 to 70 (marginal or little significant); above 70 (acceptable). Regarding the classification of usability adjectives, around 20.3 is considered worst imaginable; around 35.7 is considered bad; around 50.9 is considered fair or regular; around 71.4 is good; around 85.5 is excellent; around 90.9 is considered best imaginable^(^ 17 ^)^. After the recognition of the five quality components, the amplitude from 0 to 4 was calculated based on the answers related to each component^(^ 16 ^)^. 

This research was approved by the ethics committee of the Universidade Federal de São João Del Rei - 1.207.846, CAAE 47990215.3.0000.

## Results

In the application construction stage, the functionalities were based on 9 ITs for mobile devices traced in the literature. Of these, two were developed in the United States of America, two in China, one in Canada, Austria, Kenya, Thailand and Brazil respectively. 

In the stage of application evaluation by professors/researchers, seven judges participated in the study, with a mean age of 44.4 years, the majority of whom were female (57.1%); as for professional training, they were all nurses and had a doctoral degree. 

In the evaluation of the satisfaction questionnaire^(^ 13 ^)^ almost all the evaluated criteria obtained a mean equal to or greater than 4.0. Only the “System messages” criterion was evaluated with a 3.43 score. In the specific field for suggestions, they indicated some improvements in the application layout, such as inclusion of background image, harmonization of components with more rounded edges, inclusion of icons in some screens and addition of colors that express feeling of well-being and modernity. Regarding the messages displayed in the application, they suggested improvements in the texts that notified about overdue vaccines and in the texts detailing the vaccines, with the use of language that is less technical and easy to understand by people. Regarding the requirements of the application, they observed the need to implement a functionality for the addition of dependents’ cards, in order to facilitate the monitoring of the vaccination situation, mainly by the guardians of younger children.

**Figure 2:**
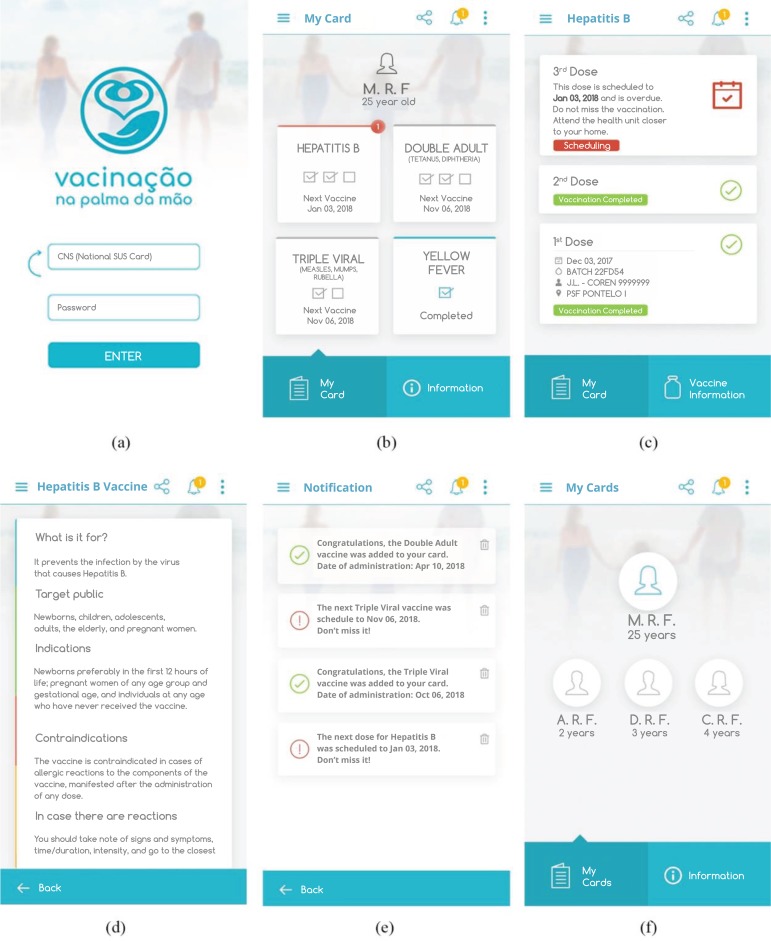
Main screens of the application: (a) access to application; (b) initial screen; (c) vaccine details; (d) vaccine information; (e) notifications; (f) dependents’ cards

All suggestions of professors/researchers were accepted, and Figure 2 shows the main graphical interfaces of the final version of the application. They are responsible for the entire user visualization and interaction process.

Figure 2a illustrates the application “Access Screen”, where the user informs the National Health Card and the password made available in the health unit. Figure 2b illustrates the “Initial Screen” after the synchronization of vaccines. This screen is composed of the “My Card” and “Information” tabs. In the “My Card” tab, users will see a list of all vaccines of their card that have been applied and, if there is any vaccine with subsequent scheduling, an informative counter of the number of scheduled doses is displayed. By clicking on a list item, users access the “Vaccine Details” screen. The “Information” tab enables obtaining information about vaccines and vaccination. The functionality of sending reminders of scheduled doses enables users to regularize their vaccination situation and, consequently, achieve vaccine coverage.

Figure 2c illustrates the “Vaccine Details” screen, displaying items such as application date, batch, vaccinator and health unit where the vaccines were administered, as well as items such as scheduling date of the next doses to be administered. On this screen, by clicking on “Vaccine Information,” users see information such as indication, contraindication, adverse events and conducts in case of post-vaccination adverse event (Figure 2d). 

The application has vaccine notification functionality. Thus, when a vaccine is registered on the SIPNI, the VPM-Sinc synchronizes the data and sends a notification to the mobile application, which directs the data to the notification screen, with description of the released dose and the scheduling information (Figure 2e). This functionality allows considering the digital vaccine card as a document to prove the users’ vaccination history, and differentiates it from other immunization applications existing in Brazil. 

For users who have dependents, the application provides the option of adding and monitoring the vaccination status of the dependent’s cards (Figure 2f).

To facilitate the sharing of vaccine card information, in case of medical appointment, the application generates the vaccine card in digital version, in Portable Document Format (PDF), for viewing and/or sharing of received and scheduled vaccines. This functionality enables the team working in the vaccination room to more easily view the person’s complete vaccinal history.

The usability evaluation using the SUS questionnaire was important to assess the interaction between users and the application. During the data collection week, we interviewed 55 people, namely 43 PHCU users and 12 health professionals. Among the users, 34 (79.1%) were aged 20-59 years; 7 (16.3%) were aged 10-19 years, and 4.6% were older than 60 years. The health professionals’ mean age was 35.1 years and they were all female. Most users (79.1%) and health professionals (75%) have used smartphones for more than three years, respectively. 

The mean SUS score obtained (90.5 ± 11.1) in the evaluation of PHCU users indicated best imaginable usability, which means that the application usability was accepted by the respondents. Based on the users’ general information, the SUS score was estimated for each characteristic such as age, sex, smartphone usage frequency, familiarity with smartphone, smartphone operating system, Internet access on the smartphone, Internet usage frequency, and whether they use any vaccination application (Table 1). 

**Table 1:** Distribution of the SUS* score related to the evaluation of the users’ general information, Pitangui, MG, Brazil, 2018

Users’ general information	SUS* score	SD†	N‡
Age			
10 to 19 years	95,4	5,3	7
20 to 59 years	89,9	12,0	34
60 years or more	83,8	1,8	2
Sex			
Female	90,2	12,4	33
Male	91,3	5,3	10
Smartphone usage frequency			
Less than a year	84,0	14,5	5
1 to 2 years	88,1	13,8	4
3 to 5 years	90,0	12,7	13
More than 5 years	92,7	8,7	21
Familiarity with Smartphone			
Yes	90,5	11,1	43
Smartphone operating system			
iOS	96,7	3,8	3
Android	90,0	11,1	37
Windows Phone	90,0	17,3	3
Internet access on the smartphone			
Yes	90,5	11,1	43
Internet usage frequency			
1 time per day	62,5	0	1
More than 1 time per day	91,1	10,3	42
Uses any vaccination application			
No	90,5	11,1	43

* SUS = System Usability Scale;

† SD = Standard Deviation;

‡ N = Number of Users

As for age group, the mean SUS score of PHCU users aged 10-19 years (95.4 ± 5.3) and 20-59 years (89.9 ± 12.0) indicated that the application usability is the best imaginable. For users aged 60 years or more, the mean SUS score of 83.8 ± 1.8 indicated excellent usability. The usability evaluation by professionals was also accepted with mean SUS score of 84.2 ± 19.4, which indicated excellent usability. 

The evaluation with PHCU users showed about the application, in decreasing order of amplitude (0-4 scale): efficiency (3.7), ease of memorization (3.7), satisfaction (3.6), minimization of errors (3.5), and ease of learning (3.5). The evaluation with professionals showed the following amplitudes: minimization of errors (3.8), efficiency (3.5), satisfaction (3.4), ease of learning (3.3), and ease of memorization (3.2).

situation are visible, such as the reemergence of diseases such as yellow fever and measles, for example.

The decrease in vaccine coverage requires the development of strategies to foster vaccination and the planning of evidence-based actions. Thus, the Vacinação na Palma da Mão advances in knowledge, in order to provide a technological tool with potential to impact on the increase of vaccine coverage and, consequently, contributing to patient safety. Health organizations need to advance toward completely recording the provided care in digital devices, enhancing patient safety. Recording data electronically, in a complete and detailed way, leads to the production of qualified information to support decisions on patient care and safety^(^ 20 ^-^ 21 ^)^.

It is important to highlight the use of applications for immunization in other countries. The ImmunizeCA application, developed in the Hospital of Ottawa (Canada), became official with functionalities of information integration and management by the country’s immunization information system^(^ 4 ^-^ 5 ^)^. The ImmunizeCA reminder function was evaluated in a study and it was found that 36% of the mothers evaluated resorted to this application functionality to monitor the vaccination records of their children, minimizing delays and, consequently, improving vaccine coverage^(^ 22 ^)^. A study conducted in San Diego found that reminder system associated with immunization records can be successful in improving vaccine coverage^(^ 23 ^)^.

A randomized clinical trial, conducted to evaluate vaccine coverage in a rural province of China, using an IT in immunization, enabled tracing children vaccination delays by generating alerts, resulting in a 17% increase in vaccination coverage in the region. Health professionals reported that the information available through the application favored the continuity of immunization activities^(^ 24 ^)^.

In Austria, the VaccApp application provides information about routine vaccinations, special vaccines, travel vaccines, among other related information. It assists in controlling and updating the vaccination status, favoring the parents’ active participation in the health of their children, constituting a tool of easy accessibility for communication in vaccination^(^ 25 ^)^. A frequently reported factor for decreased vaccination is the parents’ forgetfulness as to subsequent doses of multi-dose regimens^(^ 26 ^)^. The technological innovation built enables parents/guardians to monitor the vaccination status of the children/dependents through the functionality of adding dependents’ cards. Thus, parents or guardians will be reminded of applied and scheduled vaccines for maintenance of the dependents’ vaccine card.

 By using the application, individuals are able to track their own vaccinations, being aware of their vaccination status, solving problems in the maintenance of records^(^ 4 ^)^, and thus avoiding double vaccination and outbreaks of immunopreventable diseases^(^ 4 ^,^ 10 ^)^. In addition, the users’ access to information about their vaccination status contributes to the co-responsibility of care and the development of their autonomy aiming at disease prevention and health promotion. The use of information technologies has transformed the relations between health professionals and patients, mainly expanding access to information and sharing of information related to the health/disease/care process^(^ 27 ^)^. 

For health professionals, information technologies provide greater resolution in vaccine management, and these factors facilitate the promotion of health surveillance actions^(^ 5 ^,^ 8 ^)^. Furthermore, they support the nursing team in decision making on the administration of vaccines, which avoids waste of doses administered unnecessarily^(^ 10 ^)^. Accordingly, the decision-making process that is involved in the management of care is favored by the innovation Vacinação na Palma da Mão, because it promotes the organization and systematization of the information that will be the basis for qualified decisions about the care provided to users. 

Some applications do not ensure security of the data provided, raising concerns about the truthfulness and quality of the information. One of the mechanisms to overcome this is to have a system that validates the information provided in the applications^(^ 8 ^)^. In the developed application, the personal data security and quality are made possible by an encryption process, prior to VPM-Sinc synchronization, in addition to a user authentication system to validate the information provided and allow access to vaccinal information.

The VPM-Sinc synchronization system has an important feature of being a universal digital vaccination data communication vehicle, allowing communication with any vaccination software through the Application Programming Interface. This feature may broaden the possibilities of expanding the Vacinação na Palma da Mão mobile application by showing vaccines administered and recorded in public and private vaccination systems. It is also possible to make integrated synchronization with other vaccination systems and thus increase the completeness of the digital vaccine card of individuals, enabling the achievement of vaccination coverage goals. Such possibilities are aligned with the need to use information technologies to integrate the health care network through interoperability^(^ 28 ^)^. The objective is linking information technologies, both public or private, through a standard of information transmission. Thus, user vaccination-related information may contribute to interventions at the various points of the care network, following the user’s path through public and private health services, strengthening the process of referral and counterreferral^(^ 29 ^)^.

Among the limitations of the study, it is necessary to mention that the developed VPM-Sinc is restricted to the desktop version of SIPNI, and the application may synchronize vaccine data only from people registered in this version. The convenience and small sample limits the generalization of the usability evaluation results. It is also possible to observe another limitation regarding the need for mobile phone, restricting the research only to people who use this technology. Although the limitations were not detrimental to the research, it is expected that technological solutions are developed to correct them. 

Based on the positive evaluation of the application’s usability, one of the essential points to be developed in the continuation of this study is the installation of VPM-Sinc, considering the database of all the people registered in SIPNI, with subsequent evaluation of its impact on vaccine coverage in the municipality. 

## Conclusion

The developed and evaluated mobile app maintains the vaccine card directly in real-time synchronization with the SIPNI. For the construction of the application we listed important functionalities to improve the care in vaccination rooms and provide information to people about their vaccination status, favoring the promotion of health and the scope of vaccine coverage. 

The usability evaluation proved to be satisfactory, and the application can be considered easy to use by people and nursing professionals, with best imaginable and excellent classification, respectively. The impact of this study consists in the use of this innovation as a strategy in improving vaccine coverage and consequently in the control of immunopreventable diseases.
